# Can the delta neutrophil ındex be used as a preliminary biomarker ın the evaluation of periodontal disease: a pilot study

**DOI:** 10.1590/1678-7757-2021-0555

**Published:** 2022-03-21

**Authors:** Eda Çetin Özdemir, Emrah Bilen, Fatih M. Yazar

**Affiliations:** 1 Kahramanmaraş Sütçü İmam University Faculty of Dentistry Department of Periodontology Kahramanmaraş Turkey Kahramanmaraş Sütçü İmam University, Faculty of Dentistry, Department of Periodontology, Kahramanmaraş, Turkey.; 2 Afyonkarahisar Health Sciences University Faculty of Dentistry Department of Periodontology Afyonkarahisar Turkey Afyonkarahisar Health Sciences University, Faculty of Dentistry, Department of Periodontology, Afyonkarahisar, Turkey.; 3 Private Sular Akademi Hospital Department of General Surgery Kahramanmaraş Turkey Private Sular Akademi Hospital, Department of General Surgery, Kahramanmaraş, Turkey.

**Keywords:** Neutrophils, Lymphocytes, Periodontitis, Gingivitis

## Abstract

**Objective:**

Tissue destruction in periodontal diseases is related to inflammatory mediators in the host. However, it is unknown whether a relationship between delta neutrophil index (DNI) and neutrophil-lymphocyte ratio (NLR) in Stage 3 Grade A patients occurs. This cross-sectional study aimed to investigate the relationship between periodontal disease and DNI and NLR.

**Methodology:**

The study included 74 systemically healthy, non-smoking adults separated into 3 groups. Group 1: 26 subjects with good periodontal health, Group 2: 26 subjects with gingivitis, and Group 3: 22 subjects with Stage 3 Grade A periodontitis. After determining which group the patient will be included in, a clinical periodontal examination was made of each patient and pocket depth (PD), clinical attachment level (CAL), gingival index (GI), bleeding on probing (BOP) and plaque index (PI) parameters were measured. Venous blood samples were taken and examined with an automatic hematology analyzer for DNI, immature granulocytes (IG), NLR, C-reactive protein (CRP), procalcitonin, neutrophil count and lymphocyte count.

**Results:**

DNI, IG, CRP, and neutrophil count were observed to be highest in Group 3, followed by Group 2, and the difference between the groups in these parameters was determined to be statistically significant (p<0.001, p<0.001, p=0.046, p=0.016). DNI, IG, CRP and neutrophil count were observed to be positively correlated with periodontal parameters.

**Conclusion:**

The findings of this study support the role of DNI as a new biomarker for periodontal diseases. DNI may better reflect the systemic level of stage 3 grade A periodontitis than traditional inflammatory markers.

## Introductıon

Periodontal diseases are characterized by a series of inflammatory processes induced by micro-organisms, and result in the destruction of periodontal tissue. The most common periodontal diseases are gingivitis and periodontitis. In the formation of periodontitis, a dysbiosis in the subgingival biofilm, the host immune response and the presence of other risk factors (genetic, environmental factors) play an important role. Inflammatory cells and biofilm cause an immune and destructive response in periodontal tissues.^1^ When the acute phase of the host response becomes chronic, important systemic symptoms can emerge.^2^ Periodontitis, diabetes, and cardiovascular diseases are thought to have a common chronic inflammatory basis. Understand all inflammatory triggering factors is required to understand the onset and progression of these types of chronic diseases and develop appropriate treatments.^3^ Over time, evaluation of infection has become possible due to various cellular and circulatory biomarkers.^4^ These markers have been evaluated in disease diagnosis and follow-up in several studies on the etiopathogenesis of periodontitis. The most investigated biomarkers in biological fluids are pro-inflammatory cytokines, C-reactive protein (CRP), procalcitonin, and neutrophil count.^5,6^ Procalcitonin is a peptide precursor of the hormone calcitonin. Although its levels cannot be determined in health, studies showed that procalcitonin levels increase in inflammatory conditions due to bacterial infections. Therefore, it is considered as a marker.^7^ However, none of these are a specific biomarker for periodontitis.^1^ The delta neutrophil index (DNI) is the fraction of immature granulocytes determined by subtracting the fraction of mature polymorphonuclear leukocytes and reflects the number of immature neutrophils as a blood biomarker. It is also defined as the difference between leukocyte differentials generated in the myeloperoxidase (MPO) channel and those measured in the nuclear lobular channel provided by automated cell analyzers.^8^ DNI can be easily calculated and reported without any additional cost.^3^ Studies previously investigated DNI in diseases such as septic shock, bacteriemia,^9^ sudden cardiac arrest,^10^ renal cell carcinoma,^11^ covid-19,^12^ and gestational diabetes.^8^ Markers such as white blood cell count, absolute neutrophil count, and CRP are also effective in the prediction and prognosis of infection.^13^ Literature shows divergent results regarding neutrophil-lymphocyte ratio (NLR) and the long half-life of procalcitonin. Thus, it is necessary to determine more effective, sensitive, and relative inexpensive markers to monitor periodontal disease.

Literature lacks evaluations about the potential role of DNI in the pathogenesis of periodontal disease and its monitoring. Therefore, this study aimed to investigate the potential role of DNI in addition to the classic markers of NLR, CRP, procalcitonin, neutrophil count, and lymphocyte count in the differentiation of periodontitis according to new classification criteria^14^ in patients with gingivitis, stage 3 grade A periodontitis and a healthy control group.

## Methodology

### Study participants

This cross-sectional study included 74 systemically healthy, non-smoking individuals who attended the Periodontology Department of the Dentistry Faculty of Kahramanmaraş Sütçü İmam University, between October 1, 2020 and March 31, 2021. The study protocol was approved by the university’s Research Ethics Committee (decision no.: 06, session: 2020/18, dated: 09.30. 2020). All procedures were applied in compliance with the principles of the 2013 Helsinki Declaration. Informed consent was obtained from all the study participants. This study followed the Strengthening the Reporting of Observational studies in Epidemiology (STROBE) guidelines.^15^

The study size was calculated using the G*Power 3.1.9.4 analysis program (Heinrich-Heine-Universitat Dusseldorf, Germany). Since no sample study on the subject exists, the calculation of the power analysis was carried out according to the pilot study, conducted with 10 patients in each group. In the evaluation of the power analysis, the mean and standard deviation values of the DNI values – which are the main parameters evaluated in the study – were included. The sample size was calculated at 95% power and 5% significance level, resulting in a minimum of 19 patients per group to obtain a significant statistical value.

While forming the study population, 146 patients who applied to our clinic were invited for the study. From this total, 5 patients refused to participate in the study because they did not want to donate blood. As a result of the clinical examination, 67 people of 141 volunteers could not be included in the initially planned 3 groups. The study continued with 74 participants.

Medical and dental histories were taken from the patients, and participants were excluded if they had any systemic disease, were smokers, pregnant or breastfeeding, had used any antibiotic or anti-inflammatory drugs or received any periodontal treatment within the last 6 months.

### Medical and periodontal examinations

Patients with more than 20 teeth were evaluated clinically and radiographically. All clinical measurements were examined by a single intra-caliber clinician (E.Ç.Ö.) Full oral clinical evaluations were made with pocket depth (PD), clinical attachment level (CAL), gingival index (GI),^16^ bleeding on probing (BOP) and plaque index (PI).^17^ All the measurements were taken from 6 regions around each tooth by a clinician using a Williams-type pre-calibrated manual periodontal probe (Hu-Friedy; Chicago, IL, USA). Intra-examiner reliability was analyzed with the Kappa-Cohen test, with 0.90 and 0.88 Kappa values for PD and CAL, respectively. It was also established whether the patients had lost any tooth due to periodontitis.

### Periodontal status assessment

As a result of the clinical examinations, the systemically healthy subjects were separated into 3 groups according to the 2017 World Periodontal and Peri-Implant Diseases and Conditions Classification Workshop.^14,18^ Group 1 included 26 patients with good periodontal health, clinically healthy gingiva, BOP <10%, PD ≤3 mm, no attachment loss, no radiographic findings of alveolar bone destruction, and no history of periodontitis. Group 2 included 26 patients with gingivitis, with BOP >50%, PD≤ 3 mm, and no observation of CAL or alveolar bone loss. Group 3 included 22 patients with generalized stage 3 periodontitis, with interdental CAL ≥5 mm, PD ≥ 6 mm and radiographic bone loss extending to the middle of the root or beyond.

The patients reported the loss of more than 4 teeth because of periodontitis. In the evaluation of the spread and distribution of the disease, these patients had CAL ≥ 5 mm in ≥30% of the teeth. Appropriate measures were taken to ensure that CAL was not due to causes other than periodontitis. To estimate the progression of periodontitis, the degree of bone loss/age was determined radiographically.^19^ Radiographic bone loss was determined from the tooth showing the most severe bone loss as a percentage of root length.^19^ As bone loss %/age was <0.25, all patients were evaluated as grade A.

### Serum sampling and study variables

Venous blood samples were taken from all participants at 0800-1000 into EDTA tubes. The measurements of DNI, CRP, procalcitonin, neutrophil count, lymphocyte count, and NLR, and all the full blood analyses were performed using a calibrated automatic hematology analyzer (XN 3000, Sysmex Corpn, Kobe, Japan). NLR was evaluated manually as total neutrophil count/lymphocyte count. Also, DNI (IG%) was the ratio of the IG (immature granulocytes) count to the white blood cell (WBC) count.^20^

### Statistical analysis

Jamovi software (Version 1.6.23) was used for statistical analysis. Data were stated as mean ± standard deviation (SD) values. Normality of distribution was checked with Shapiro Wilk test. Due to non-normal distribution, Kruskal-Wallis analysis was conducted to detect the differences between groups. Dwass-Steel-Critchlow-Fligner (DSCF) was conducted for multiple comparisons analysis. Gender differences between groups were evaluated using Chi-squared test. The relationship between periodontal and inflammatory variables were analyzed with Spearman’s correlation test. The optimal cutoff value for each variable in the diagnosis of periodontitis was determined with Receiver Operating Characteristic (ROC) curve analysis. The areas under the curve (AUC) were stated in a 95% confidence interval (CI). Statistical significance was considered when p<0.05.

## Results

**Figure 1:**
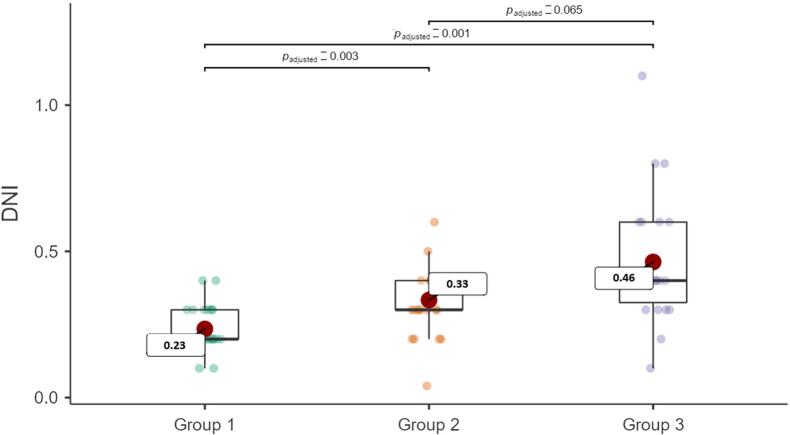
Intergroup difference in DNI value

Table 1 shows the clinical characteristics and inflammatory parameters of all patients included in the study. Age ranged between 18 and 65 years (28 males, 46 females). The mean age of the individuals in the periodontitis group was higher than the other groups (p=0.012), and gender difference was statistically significant (p=0.033). In the evaluation of the DNI of the groups, DNI values (Figure 1) were significantly higher in the periodontitis group than control group (p<0.001, respectively).

**Table 1:** The clinical characteristics and inflammatory parameters of all the patients

Characteristic	Group 1	Group 2	Group 3	p
	(n=26)	(n=26)	(n=22)	
**Demographic variables**				
Age	35.53±8.45^b^	35.07±11.30^ab^	38.36±7.02^a^	0,012
Sex (male/female) n	8/18	6/20	14/8	0,033
**Inflammatory variables**				
CRP	3.51±1.36^b^	4.39±2.44^b^	6.26±5.73^a^	0,046
Procalcitonin	0.038±0.014^a^	0.046±0.023^a^	0.060±0.064^a^	0,316
IG	0.015±0.005^b^	0.028±0.009^a^	0.036±0.016^a^	<0.001
DNI	0.23±0.07^b^	0.33±0.11^a^	0.46±0.22^a^	<0.001
Neutrophil count 109	3.96±1.03^b^	4.70±1.45^ab^	4.96±1.28^a^	0,016
Lymphocyte	2.25±0.51^a^	2.50±0.53^a^	2.40±0.71^a^	0,187
NLR	1.85±0.67^a^	1.96±0.78^a^	2.22±0.85^a^	0,156
**Periodontal parameters**				
PI	0.55±0.16^c^	1.91±0.20^b^	2.40±0.20^a^	<0.001
GI	0.61±0.18^c^	1.86±0.32^b^	2.54±0.18^a^	<0.001
PD	1.57±0.22^c^	2.49±0.48^b^	4.52±0.60^a^	<0.001
BOP	6.5±1.53^c^	19,25±12.84^b^	79.70±5.39^a^	<0.001
CAL	0^c^	2.21±0.66^b^	5.14±0.49^a^	<0.001

Data are expressed as the mean ±SD or n (%), unless otherwise noted. Kruskal-Wallis test and Dwass-Steel-Critchlow-Fligner was used for all the parameters, except gender. Gender differences between groups were calculated using chi-squared test. Different superscript letters indicate significance in a row (p<0.05). CRP(C-reactive protein), IG(immature granulocytes), DNI(delta neutrophil index), NLR(neutrophil-lymphocyte ratio), PI(plaque index), GI(gingival index), PD(pocket depth), BOP(bleeding on probing),CAL(clinical attachment level)

**Figure 2:**
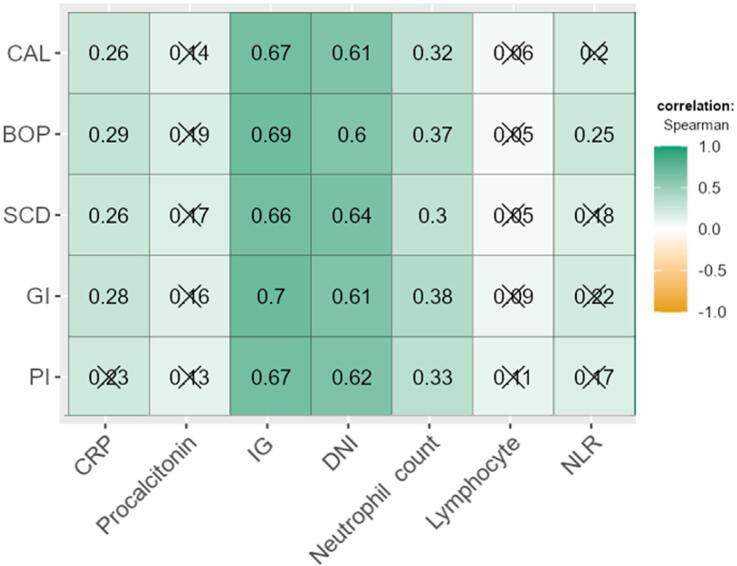
Evaluation of the relationship between periodontitis and hematological parameters by correlation analysis

The correlation analyses between hematological and periodontal parameters showed a positive correlation between periodontal parameters and the DNI, IG, neutrophil count and CRP (except PI) values. Figure 2 shows the factors related to periodontal parameters.

Correlation matrix boxes: green and orange boxes indicate positive and negative correlations, respectively (Colors tones indicate the strength of the correlation). Boxes without “X” indicate significance (p<0.05). Numbers on the boxes are spearmen correlation coefficients.

**Figure 3:**
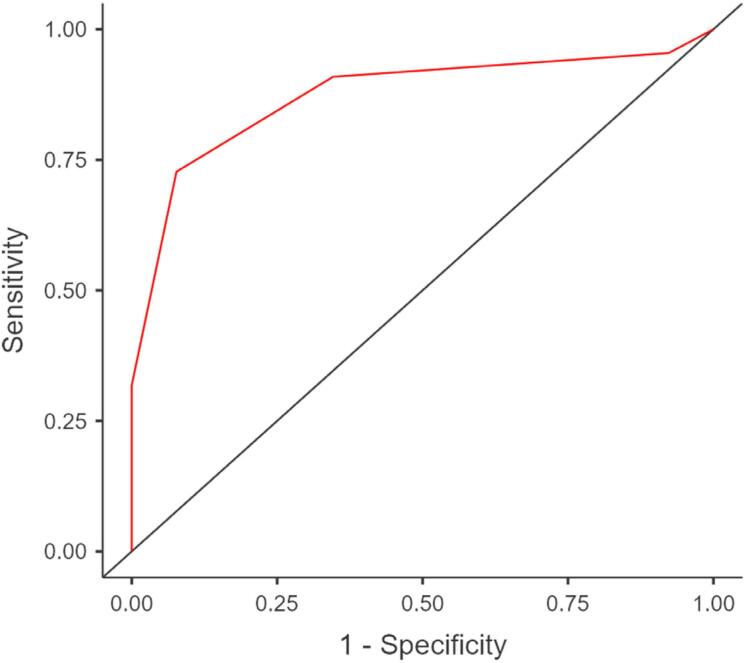
Specificity sensitivity graph

In the ROC analysis, the optimal 0.25 cutoff value for the DNI value in the diagnosis of periodontal disease showed 91% sensitivity and 65% specificity. DNI showed relation to periodontal parameters in the correlation analysis. Figure 3 shows the sensitivity and specificity values obtained by the ROC analysis.

## Discussion

This study demonstrated that DNI value is closely related to periodontal disease. In contrast to NLR, procalcitonin, CRP, neutrophil count, lymphocyte count, and other markers, DNI is a new biomarker with which many clinicians are not familiar due to its lack of research in periodontal diseases. This study aimed to evaluate whether DNI was more sensitive and specific than other classic inflammatory markers at various stages of periodontal diseases.

Park et al.^13^(2020) evaluated if DNI was a predictive marker in the prognosis of chronic obstructive pulmonary disease and reported that increased DNI levels were significant. Ahn, et al.^9^ (2014) also reported a positive relationship between DNI and bacteremia in immunosuppressed children. A systematic review that investigated the role of DNI in infected patients concluded that DNI was a potential diagnostic tool and should be used more widely.^3^ Despite several reports that DNI is better than other inflammatory markers in the diagnosis of infection, studies lack examinations whether DNI is valid in other chronic inflammatory diseases such as periodontal diseases, or whether the general diagnostic accuracy of DNI is comparable to other traditional markers.

When this study considered the inflammatory character of periodontal diseases, it was assumed that DNI would increase in patients with gingivitis and periodontitis. In a study that examined the relationship between DNI and gestational diabetes, Uysal et al reported a positive relationship.^8^ Studies also reported that DNI level was increased in pathological conditions such as sepsis, bacteremia, and pre-eclampsia.^20^ Furthermore, investigations analyzing pathophysiological background of periodontal diseases showed that bacteria and their products cause bone destruction and various inflammatory mediators are expressed from bone. Therefore, pathological conditions activated chemotactic factors and cytokines may be associated with an acute phase response which stimulates bone marrow to express immature granulocytes into the circulation.^21^ In our study, a statistically significant difference was determined between the groups regarding the DNI level.

Moreover, the half-life of DNI is 3 hours, which is much shorter than the 24–30 hours of procalcitonin.^22^ A shorter half-life more easily reflects the infection status of the patient and is useful during follow up for the evaluation of the therapeutic efficacy of the treatment applied. In our study, while a significant difference was determined between the groups in respect of DNI, no significant difference was found for procalcitonin. This suggests that DNI could be a more effective precursor than procalcitonin in reflecting the systemic inflammatory load. In another study that evaluated procalcitonin and DNI as predictive and prognostic biomarkers with ROC curve analysis, it was concluded that DNI had better general diagnostic accuracy.^3^

NLR seems to reflect the balance between natural and acquired immune mechanisms.^23^ In inflammatory diseases, NLR is a marker of both inflammatory load (neutrophil count) and regulatory mechanisms (lymphocyte count).^24^ Therefore, NLR may reflect the first natural immune mechanisms that trigger adaptive immune mechanisms which cause periodontal destruction.^25^ A previous study reported a positive relationship between NLR and chronic periodontitis, and NLR was a sensitive and specific biomarker for periodontitis.^26^ However, other studies reported conflicting results. A recent study stated that NLR was not affected by the severity of periodontal disease or the glycemic level.^24^ Studies also evaluated the role of NLR in periodontitis patients with risk factors such as diabetes, hyperlipidemia, or obesity.^27^ According to that study, different risk factors in periodontitis patients showed no significant difference. Unlike that study, our study included patients who were systemically healthy, those with gingivitis and those with stage 3 grade A periodontitis, and although we observed increased NLR level in the periodontitis group compared to the other groups, no statistically significant difference was determined. Therefore, it seemed that this parameter is not a sufficiently sensitive and specific biomarker in a patient group with low-level chronic inflammation such as diabetes and periodontitis.

Investigations indicates that CRP is a classic biomarker related to periodontitis.^28^ In a previous study that defined the association between inflammation and CRP and other inflammation markers, 3 groups were formed and — according to the results — if CRP was >3, there was no significant difference in the procalcitonin and NLR values.^29^ Consistent with these findings, in our study groups, CRP values were >3, and showed no difference in respect of NLR and procalcitonin.^21^ In general, literature reported a positive relationship between inflammation severity and periodontal diseases.^1^ Our results shows that CRP was highest in Group 3, and the difference from the other groups was statistically significant. In addition, we compared the neutrophil and lymphocyte values between the groups, and consistent with the literature,^4^ only the neutrophil count was found to be statistically significantly different between the groups.

In the evaluation of our results, the cross-sectional design should be considered as a limitation. The local examination of these type of parameters may provide more sensitive results in the evaluation of periodontal disease. Further long-term studies of larger populations to evaluate the effect of periodontal treatment on these parameters are necessary.

## Conclusion

When the results of this study are examined in detail, it may be said that DNI is associated with periodontal diseases. Furthermore, the results showed consistency with literature in respect of CRP and the neutrophil count. DNI could be considered to be a promising new marker that requires further research in periodontal diseases. Although other studies show that DNI increases in acute infections, our study found that it may also increase in chronic diseases such as periodontitis.
